# Liver Cirrhosis among Young Adults Admitted to the Department of Gastroenterology in a Tertiary Care Centre: A Descriptive Cross-sectional Study

**DOI:** 10.31729/jnma.8016

**Published:** 2023-02-28

**Authors:** Mohan Bhusal, Rahul Pathak, Brindeswari Kafle Bhandari, Anurag Jha, Rabin Hamal, Dinesh Koirala, Manoj Lamsal, Pradip Kumar Kafle

**Affiliations:** 1 Department of Gastroenterology, Tribhuwan University Teaching Hospital, Maharajgunj, Kathmandu, Nepal

**Keywords:** *ascites*, *liver cirrhosis*, *prevalence*

## Abstract

**Introduction:**

Cirrhosis in young adults is an important health problem worldwide and is a common disease. Patients usually present late in a decompensated state with varied complications. However, national data on the exact burden of the disease is lacking. The aim of this study was to find out the prevalence of liver cirrhosis among young adults admitted to the Department of Gastroenterology in a tertiary care centre.

**Methods:**

A descriptive cross-sectional study was done among patients admitted to the Department of Gastroenterology in a tertiary care centre between 25 November 2021 to 30 November 2022 after receiving ethical approval from the Institutional Review Committee [Reference number: 227(6-11) E2-078/079]. Convenience sampling was done. Point estimate and 95% Confidence Interval were calculated.

**Results:**

Among 989 patients, liver cirrhosis in young adults was seen in 200 (20.22%) (18.12-22.32, 95% Confidence Interval). Chronic alcohol use was the primary cause of cirrhosis seen in 164 (82%) cases. The most typical presenting symptom was abdominal distension seen in 187 (93.50%) patients. The most frequent complication was ascites seen in 184 (92%) patients. The most frequent endoscopic finding was gastro-oesophagal varices seen in 180 (90%) patients. There were 145 (72.50%) men and 55 (27.50%) women.

**Conclusions:**

The prevalence of liver cirrhosis in young adults was found to be lower than the other studies done in similar settings.

## INTRODUCTION

Liver cirrhosis refers to a disorder that alters the overall typical architecture of the liver. Globally, the majority of instances are ascribed to non-alcoholic fatty liver disease, viral hepatitis, or excessive alcohol usage.^1^ Depending on the aetiology and whether portal hypertension or hepatocellular damage predominates, the clinical appearance of cirrhosis differs. However, even in the absence of any clear clinical symptoms, substantial liver damage may be present.^2^

Various causes of cirrhosis in adults have been studied but the aetiology of cirrhosis in young adults less than or equal to 40 years has not been well studied, and the incidence of cryptogenic cirrhosis remains unknown.^3^ Early interventions and preventions are required to stabilize disease progression and to avoid or delay clinical decompensation and the need for liver transplantation.^4^

The aim of this study was to find out the prevalence of liver cirrhosis among young adults admitted to the Department of Gastroenterology in a tertiary care centre.

## METHODS

A descriptive cross-sectional study was done among young adults admitted to the Department of Gastroenterology in Tribhuvan University Teaching Hospital between 25 November 2021 to 30 November 2022 after receiving ethical approval from the Institutional Review Committee of same institute [Reference number: 227(6-11)E2-078/079]. All patients admitted to the Gastroenterology ward of the hospital aged >18 and ≤40 years were included in the study. Patients who do not give informed consent were excluded from the study. Informed consent was signed and confidentiality of the information was ensured. Convenience sampling was done. The sample size was calculated by using following formula:


$$\begin{array}{ll} \text{n} & ={\text{Z}}^{2}\times \frac{\text{p}\times \text{q}}{{\text{e}}^{\text{2}}}\\ & =1.96^{2}\times \frac{0.50\times 0.50}{0.04^{2}}\\ & =601 \end{array}$$

Where,

n = minimum required sample size
Z = 1.96 at a 95% Confidence Interval (CI)
p = prevalence taken as 50% for maximum sample size calculation
q = 1-p
e = margin of error, 7%

The calculated minimum required sample size 601. However, 989 patients were included in the study.

Each patient was subjected to a detailed clinical history regarding the duration of illness and symptoms. Predetermined proforma was used as the tool for data collection. All patients were subjected to detailed clinical and laboratory data including demographics, and history of alcohol consumption, medications, substance abuse, and other systemic diseases. Various biochemical studies like alkaline phosphatase (ALP), aspartate aminotransferase (AST), alanine transaminase (ALT), serum total globulin/ gamma globulins, serology like antinuclear antibody (ANA), anti-smooth muscle antibody (ASMA), antimitochondrial Aantibody (AMA), immunoglobulin A (IgA), tissue transglutaminase (tTG), liver-kidney microsomal (LKM-1), viral hepatitis markers, HLA-DR3 or DR4) and abdominal ultrasound was done for liver and spleen size, parenchymal echogenicity, portal vein diameter, and ascites. Serum ceruloplasmin, urinary copper levels and slit lamp examination for the Kayser-Fleischer ring were done when indicated.^5^

Each patient had undergone upper gastrointestinal (UGI) endoscopy and diagnostic findings were documented. Child-Turcotte-Pugh (CTP) score and Model for End Stage Liver Disease (MELD) scores were calculated for all the patients. Information was gathered using a standardized proforma.

Data collected were entered and analyzed using IBM SPSS Statistics version 20.0. Point estimate and 95% CI were calculated.

## RESULTS

Among 989 patients, liver cirrhosis among young adults was seen in 200 (20.22%) (18.12-22.32, 95% CI). In a total of 200 cases, liver cirrhosis was seen in 145 (72.50%) men and 55 (27.5%) women. The participants ranged in age from 18 to 40 years, with mean age of 28.92±5.73 years. A total of 68 (34%) Brahmins made up the majority of the study group, followed by 42 (21%) Khas, 25 (12.50%) Newar, 20 (10%) Madheshi, and 6 (3%) Tharus.

There were 73 (36.50%) farmers made up the research group, which also included 56 (28%) retired people, 37 (18.50%) people who worked for the government, and 34 (17%) housewives. In this study 113 (56.50%) patients were from rural areas, while 87 (43.50%) patients were from metropolitan areas. A total of 80 (40%) patients were from a moderate socioeconomic class, 70 (35%) from a lower one and just 50 (25%) from a higher one.

**Figure 1:**
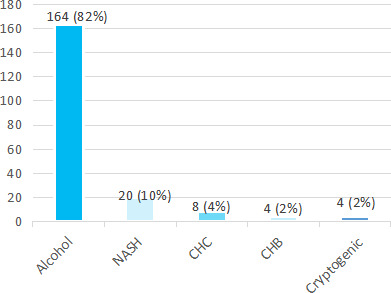
Suspected causes in patients with cirrhosis (n= 200).

Chronic alcohol use was the primary cause of cirrhosis in 164 (82%) patients. Other causes were non-alcoholic steatohepatitis (NASH) and chronic viral hepatitis seen in 20 (10%) cases and 12 (6%) cases respectively. The remaining cases, 4 (2%) were labelled as cryptogenic (Figure 1).

Abdominal distension was the most frequent manifestation, occurring in 187 (93.50%) cases, followed by anorexia 140 (70%), fatigue 120 (60%), and vomiting 104 (52%). Ascites was clinically evident in 184 (92%) individuals. There were 108 (54%) individuals who had upper gastrointestinal bleeding. The other typical signs were icterus, followed by pallor, pedal edema, and hair loss over the body (Table 1).

**Table 1:** Symptomatology at presentation (n= 200).

Signs and symptoms	n (%)
Abdominal distension	187 (93.50)
Anorexia	140 (70)
Fatigue	120 (60)
Vomiting	104 (52)
Dizziness	64 (32)
Fever	50 (25)
Altered sensorium	36 (18)
Oliguria	12 (6)
Ascites	184 (92)
UGI bleed	108 (54)
Icterus	148 (74)
Pallor	144 (72)
Pedal oedema	120 (60)
Loss of body hair	118 (59)
Spider naevi	86 (43)
Palmar erythema	48 (24)
Parotid enlargement	52 (26)
Dyspnea	40 (20)

There were 108 (54%) individuals who had UGI bleeding. The most frequent endoscopic finding was gastro-oesophagal varices, which were discovered in 180 (90%)patients, followed by portal gastropathy in 150 (75%) patients, peptic ulcers in 15 (7.50%) patients, gastro-duodenitis in 4 (2%) patients, Mallory Weiss tears in 20 (10%) patients and GI malignancies in 2 (1%) patients.

**Figure 2:**
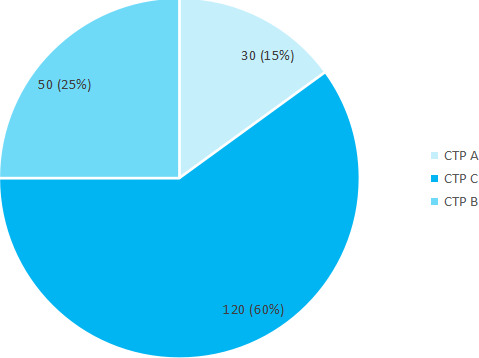
Child-Turcotte-Pugh score of patients with cirrhosis (n= 200).

The participants were divided into groups based on their CTP classifications. Most cases belong to CTP C 120 (60%) patients (Figure 2).

The most common complications was ascites seen in 184 (92%) of the patients. Hepatic encephalopathy (HE) was seen in 36 (18%) cirrhotic patients, followed by spontaneous bacterial peritonitis (SBP) in 26 (13%) patients, and hepatorenal syndrome (HRS) in 22 (11%) patients.

## DISCUSSION

The prevalence of liver cirrhosis among young adults was 20.22% which was high when compared to a similar study done in other tertiary care centres of Nepal,^6^ but lower to the similar studies done in Nepal.^7,8^

In the current investigation, alcoholic liver disease was the most prevalent aetiology of cirrhosis and was found in 164 (82%) individuals which was similar to other studies.^4,9,10^ The most prevalent cause of cirrhosis in Nepal is chronic alcohol use. Therefore, cirrhosis cases are increasingly being detected in young people, as was shown in the current research, which may be related to early alcohol consumption and dependency. The prevalence of alcohol use, misuse, and dependency among the younger population is increasing, which may be the cause of this condition.

In this study, 184 (92%) individuals had ascites followed by pallor in 144 (72%) and pedal edema in 120 (60%), icterus was seen in 148 (74%) patients. In this research, 108 (54%) patients had upper gastrointestinal bleeding. These results were similar to other studies.^4,9^ In the current study, the most frequent finding on UGI endoscopy was gastro-oesophageal varices, which were observed in 180 (90%) patients, followed by portal gastropathy in 150 (75%) patients which was similar to other studies.^9^

The mean age of the patients from our study was similar to these studies.^9^ With regards to the gender-wise distribution of the patients, our study showed that ALD was more predominant in males which is similar to other studies.^4,9,10^ The increased prevalence of ethanol use among males compared to women is most likely the cause of the male preponderance over female in all investigations. Additionally, there may be disparities in how the two sexes seek medical attention.

In the present research, a total of 113 (56.5%) patients came from rural regions which was less compare to other studies.^9^ The CTP score of our study was similar to the studies carried out at other tertiary centre.^9^ In our investigation, ascites, which was discovered in 184 (92%) patients, was determined to be the most typical complication of cirrhosis at presentation, followed by UGI bleed in 108 (54%) patients. Rebleeding was seen in 33 (16.5%) patients. Hepatic encephalopathy 36 (18%), SBP 26 (13%), and HRS 22 (11%) followed. According to one of the study, the most frequent complications were ascites in 78.6% of patients, variceal bleeding in 43.4%, hepatic encephalopathy in 21.6%, SBP in 4.2%, HRS in 2.7%, HCC in 1.3%, hypersplenism in 0.4%, and sepsis in 12.8% of patients.^9^ These findings were consistent with those from our study. In some investigations, SBP incidences between 10% and 30% higher than ours have been reported.^10-12^ According to a recent study, hospitalized patients had a prevalence of SBP of 24.7% and 34.9%, respectively.^13,14^

The results of the study cannot be generalised as the population under study is limited to patients admitted to one tertiary care centre. Also, because of the descriptive nature of this study, an association between exposure and outcome cannot be made in this study design and risk factors cannot be made out. A larger study conducted at different centres should be conducted to better understand the exact burden of liver cirrhosis in young adults.

## CONCLUSIONS

The prevalence of liver cirrhosis in young adults in our study was found to be lower than in studies done in similar settings. People need to be made aware of the negative consequences of frequent alcohol use. Early diagnosis of viral hepatitis and alcoholic liver illnesses provides survival advantages, and their treatment may lessen the burden of cirrhosis. In instances of cirrhosis that have already progressed, necessary management and therapy, the avoidance of complications, and routine monitoring and follow-ups may all lower morbidity and death.
